# Relevance of instrumented gait analysis in the prediction of the rebound phenomenon after guided growth intervention

**DOI:** 10.1038/s41598-024-66169-9

**Published:** 2024-07-11

**Authors:** Felix Stief, Jana Holder, Sebastian Braun, Marco Brenneis, Stefan van Drongelen, S. Kimberly Byrnes, Frank Layher, Chakravarthy U. Dussa, Andrea Meurer, Harald Böhm

**Affiliations:** 1 Department of Trauma Surgery and Orthopedics, University Hospital, Goethe University Frankfurt, Marienburgstraße 2, 60528 Frankfurt/Main, Germany; 2 Berufsgenossenschaftliche Unfallklinik Frankfurt/Main, Friedberger Landstraße 430, 60389 Frankfurt/Main, Germany; 3 Orthopaedic Children’s Hospital, Bernauer Straße 18, 83229 Aschau I. Chiemgau, Germany; 4 Institute for Conservative and Rehabilitative Orthopedics, Department of Sports and Health Sciences, Technical University of Munich, Georg-Brauchle-Ring 60/62, 80992 Munich, Germany; 5 Orthopedic Department of the Waldkliniken Eisenberg, Orthopaedic Professorship of the University Hospital Jena, Klosterlausnitzer Straße 81, 07607 Eisenberg, Germany; 6 Department of Trauma and Orthopaedic Surgery, Friedrich-Alexander-Universität Erlangen-Nürnberg, Rathsberger Str. 57, 91054 Erlangen, Germany; 7 PFH Private University of Applied Sciences, Weender Landstraße 3-7, 37073 Göttingen, Germany; 8 Present Address: Department of Sport and Exercise Science, University of Salzburg, Schlossallee, 5400 Hallein, Salzburg, Austria; 9 Present Address: Center for Musculoskeletal Surgery, University Hospital, Corporate Member of Freie Universität Berlin and Humboldt-Universität Zu Berlin, Charité –Universitätsmedizin Berlin, Rahel-Hirsch-Weg 5, 10117 Berlin, Germany; 10 Present Address: Medical Park St. Hubertus Klinik, Sonnenfeldweg 29, 83707 Bad Wiessee, Germany

**Keywords:** Gait analysis, Leg malalignment, Temporary hemiepiphysiodesis, Rebound, Dynamic knee joint loading, Genu valgum, Mechanical engineering, Paediatric research, Outcomes research

## Abstract

Predictors of rebound after correction of coronal plane deformities using temporary hemiepiphysiodesis (TH) are not well defined. The following research questions were tested: (1) Is the dynamic knee joint load useful to improve rebound prediction accuracy? (2) Does a large initial deformity play a critical role in rebound development? (3) Are BMI and a young age risk factors for rebound? Fifty children and adolescents with idiopathic knee valgus malalignment were included. A deviation of the mechanical femorotibial angle (MFA) of ≥ 3° into valgus between explantation and the one-year follow-up period was chosen to classify a rebound. A rebound was detected in 22 of the 50 patients (44%). Two predictors of rebound were identified: 1. reduced peak lateral knee joint contact force in the first half of the stance phase at the time of explantation (72.7% prediction); 2. minor initial deformity according to the MFA (70.5% prediction). The best prediction (75%) was obtained by including both parameters in the binary logistic regression method. A TH should not be advised in patients with a minor initial deformity of the leg axis. Dynamic knee joint loading using gait analysis and musculoskeletal modeling can be used to determine the optimum time to remove the plates.

## Introduction

Guided growth using temporary hemiepiphysiodesis (TH) is a common procedure for the treatment of coronal plane deformities around the knee in children and adolescents with sufficient residual growth^1^. Despite the minimally invasive nature, TH has been associated with prolonged postoperative pain, reduced mobility and activity in the operated knee between five weeks and six months in up to 35% of the patients^2,3^.

The incidence of recurrence (rebound phenomenon) of the initial axial deformity after successful correction of the leg axis and removal of the implant varies considerably, and predictors of rebound are not well defined and understood^4^. The validity of most studies is affected by several limitations, including a non-standardized follow-up after plate removal, heterogeneous patient groups with a small number of cases, and missing information on the definition of rebound^4^. The following risk factors for the occurrence of rebound were discussed: large initial deformities^5^, higher^6^ or lower body mass index (BMI)^7^ in patients with idiopathic valgus malalignment. Moreover, young age at the beginning of treatment with substantial remaining growth potential after implant removal is most frequently reported in the literature^5–8^. However, after growth termination in adulthood, recurrence of the initial deformity can occur after axis correction following high tibial osteotomy^9^. Thus, the presence of residual growth after implant removal cannot be the only factor for recurrence, which necessitates the exploration of other predictors.

The mechanical load at the knee joint during gait can affect the development of the leg axis^10,11^. According to the Hueter-Volkmann law^12^, unilateral changes in compressive forces cause asymmetrical growth of a joint. The chondral modeling theory^10^ suggests that physiological loading stimulates growth, while loads outside this range, either higher or lower, will lead to suppression. Instrumented gait analysis, in combination with musculoskeletal (MSK) modeling, can be used as an indirect method to assess the dynamic knee joint loading. The external knee adduction moment (KAM) is the most used measure of joint loading and has an important relationship with the initiation and progression of knee osteoarthritis (OA)^13,14^. MSK modeling can reveal compressive forces in the lateral and medial compartments, which may be closer to the actual forces acting at the knee joint^15^.

The objective of the present prospective study was to identify predictors of rebound at the time of implantation and explantation of the plates in a homogeneous group of children and adolescents with idiopathic knee valgus malalignment following a TH. In particular, we primarily performed this study to answer the following questions: (1) Is the mechanical load at the knee joint during gait useful to improve rebound prediction accuracy? (2) Does a large initial deformity play a critical role in rebound development? (3) Are BMI and a young age at the beginning of treatment with substantial remaining growth potential after implant removal risk factors for the occurrence of rebound?

## Results

### Radiological examination

Considering the initial deformity, both the radiographic MAD (*p* = 0.011) and MFA (*p* = 0.006) were significantly more pronounced in the group without an occurrence of rebound phenomenon. In contrast, no significant group differences were detected in radiographic parameters at the time of explantation (Table 1).

**Table 1:** Group differences (no rebound, rebound) in study population characteristics and radiographic parameters (*n* = 50) at the time of implantation and explantation of the plates.

Parameter	No Rebound	Rebound	*p*-Value
Number of subjects	28	22	
Sex [female/male]	9(32%)/19(68%)	8(36%)/14(64%)	0.797
Time of implantation of the plates			
Anthropometric parameters			
Age [years/months]	13/3 (1/2)	12/8 (1/0)	0.184
Height [m]	1.68 (0.10)	1.64 (0.10)	0.211
Body mass index [kg/m^2^]	22.91 (3.50)	22.58 (3.79)	0.768
X-ray parameters			
MAD [mm]	-23.0 (7.2)	-17.6 (5.6)	**0.011**
MFA [°]	-6.6 (1.7)	-5.1 (1.7)	**0.006**
Time of explantation of the plates			
Anthropometric parameters			
Age [years/months]	14/3 (1/1)	13/6 (1/1)	0.054
Height [m]	1.73 (0.11)	1.69 (0.10)	0.200
Body mass index [kg/m^2^]	23.84 (4.12)	23.56 (4.16)	0.825
X-ray parameters			
MAD [mm]	-1.8 (7.5)	2.4 (6.0)	0.056
MFA [°]	-0.5 (2.1)	0.6 (1.8)	0.054
Implant localization			
Medial distal femur [number]	18	17	0.347
Medial proximal tibia [number]	4	3	
Medial distal femur and medial proximal tibia [number]	6	2	
Duration of guided growth and growth characteristics of the study population			
Duration of guided growth [months]	12.1 (5.1)	9.9 (2.4)	0.068
Growth between implantation and explantation of the plates [cm]	5.1 (1.9)	5.0 (1.7)	0.818
Residual growth—from time of explantation to one-year follow-up [cm]*	2.5 (1.4–4.2)	5.9 (3.5–7.4)	**0.009**

Normally distributed data: Mean with standard deviation in parenthesis. *Not normally distributed data: Median with interquartile range in parenthesis. MAD: mechanical axis deviation, medial deviation (varus) is depicted as positive and lateral deviation (valgus) as negative values; MFA: mechanical femorotibial angle, varus malalignment is depicted as positive angles and valgus malalignment as negative angles. Significant differences are bold printed.

**Figure 1:**
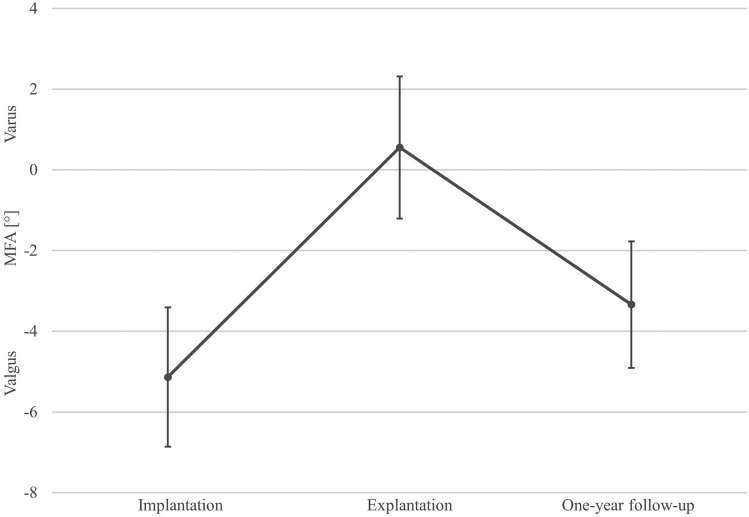
Mean and standard deviation of the mechanical femorotibial angle (MFA) changes over time of the rebound group (*n* = 22).

The MFA in the rebound group increased from − 5.1 ± 1.7° valgus malalignment at the time of implantation to a neutral alignment of 0.6 ± 1.8° at the time of explantation and decreased again to − 3.3 ± 1.6° valgus malalignment at the one-year follow-up (Fig. 1).

### Kinematics and mechanical load at the knee joint during gait

Of the 50 included patients, 22 developed a rebound at the one-year follow-up period after implant removal, indicating a rebound rate of 44%. The three groups did not show any significant differences in spatiotemporal kinematic gait parameters (e.g., walking speed) neither at the time of implantation nor at the time of explantation (Table 2). At the time of implantation, the external KAMs (both peaks and impulse) were significantly lower in the no rebound group compared to controls. No significant differences were detected between the rebound group and controls. The peak medial knee joint contact forces were significantly lower in both patient groups compared to controls. The peak lateral knee joint contact forces were significantly higher in the no rebound group compared to the rebound group and controls (Table 2).

**Table 2:** Group differences (no rebound, rebound, controls) in study population characteristics and gait parameters at the time of implantation and explantation of the plates.

Parameter	No Rebound	Rebound	Controls	*p*-Value
Number of subjects	28	22	15	
Sex [female/male]	9(32%)/19(68%)	8(36%)/14(64%)	6(40%)/9(60%)	0.732
Time of implantation of the plates				
Anthropometric parameters				
Age [years/months]	13/3 (1/2)	12/8 (1/0)	13/5 (1/4)	0.373
Height [m]	1.68 (0.10)^b^	1.64 (0.10)	1.62 (0.10)^b^	**0.037**
Body mass index [kg/m^2^]	23.40 (20.36–25.45)^b^	23.40 (20.80–25.20)^c^	17.70 (16.60–20.70)^b,c^	** < 0.001**
Spatiotemporal gait parameters				
Walking speed [m/s]	1.23 (0.13)	1.18 (0.15)	1.23 (0.12)	0.183
Normalized walking speed [v/$\sqrt{g\times leg length}$]	0.41 (0.03)	0.40 (0.06)	0.43 (0.04)	0.261
Step length [cm]	65.3 (5.8)	61.5 (7.0)	62.0 (6.2)	0.094
Normalized step length [step length/leg length]	0.71 (0.04)	0.69 (0.07)	0.73 (0.06)	0.132
Step width [cm]*	10.5 (9.9–12.7)	11.4 (9.9–13.7)	9.1 (8.6–10.9)	0.085
External knee joint moments in the frontal plane [Nm/kg]				
Peak knee adduction moment 1st half of stance*	0.21 (0.17–0.30)^b^	0.26 (0.22–0.41)	0.42 (0.35–0.51)^b^	** < 0.001**
Peak knee adduction moment 2nd half of stance	0.20 (0.10)^b^	0.26 (0.10)	0.32 (0.09)^b^	**0.002**
Knee adduction moment impulse (area under the curve)	26.49 (19.70)^b^	41.22 (17.06)	56.41 (14.94)^b^	** < 0.001**
Peak knee joint contact forces [N/(kg × m/s^2^)]				
Medial knee joint contact force 1st half of stance*	1.70 (1.31–1.82)^b^	1.71 (1.58–1.88)^c^	2.07 (1.87–2.40)^b,c^	** < 0.001**
Medial knee joint contact force 2nd half of stance*	2.14 (1.78–2.46)^b^	1.96 (1.78–2.88)^c^	2.80 (2.43—3.37)^b,c^	**0.007**
Lateral knee joint contact force 1st half of stance	1.55 (0.24)^a,b^	1.33 (0.33)^a^	1.14 (0.16)^b^	** < 0.001**
Lateral knee joint contact force 2nd half of stance	1.86 (0.32)^a,b^	1.60 (0.39)^a^	1.40 (0.23)^b^	** < 0.001**
Time of explantation of the plates				
Anthropometric parameters				
Age [years/months]	14/3 (1/1)	13/6 (1/1)	13/5 (1/4)	0.131
Height [m]	1.73 (0.11)^b^	1.69 (0.10)^c^	1.62 (0.10)^b,c^	** < 0.001**
Body mass index [kg/m^2^]	24.93 (20.73–26.95)^b^	24.50 (21.40–26.10)^c^	17.70 (16.60–20.70)^b,c^	** < 0.001**
Leg alignment				
MFA [°]—non-invasive marker-based approach	− 1.0 (2.1)	− 0.0 (1.7)	− 1.0 (1.3)	0.165
Spatiotemporal gait parameters				
Walking speed [m/s]	1.22 (0.12)	1.17 (0.14)	1.23 (0.12)	0.178
Normalized walking speed [v/$\sqrt{g\times leg length}$]	0.40 (0.04)	0.39 (0.04)	0.43 (0.04)	0.161
Step length [cm]	64.7 (7.6)	62.0 (5.7)	62.0 (6.2)	0.164
Normalized step length [step length/leg length]	0.72 (0.05)	0.69 (0.06)	0.73 (0.06)	0.078
Step width [cm]*	10.5 (9.9–12.7)	11.4 (9.9–13.7)	9.1 (8.6–10.9)	0.945
External knee joint moments in the frontal plane [Nm/kg]				
Peak knee adduction moment 1st half of stance	0.53 (0.17)	0.50 (0.13)	0.44 (0.13)	0.228
Peak knee adduction moment 2nd half of stance	0.43 (0.12)^b^	0.43 (0.09)^c^	0.32 (0.09)^b,c^	**0.004**
Knee adduction moment impulse (area under the curve)	71.22 (19.06)^b^	74.29 (17.98)^c^	56.41 (14.94)^b,c^	**0.012**
Peak knee joint contact forces [N/(kg × m/s^2^)]				
Medial knee joint contact phase 1st half of stance	2.13 (0.34)	2.06 (0.32)	2.15 (0.36)	0.723
Medial knee joint contact phase 2nd half of stance	2.90 (0.62)	2.87 (0.66)	2.78 (0.59)	0.836
Lateral knee joint contact force 1st half of stance	1.02 (0.22)^a^	0.83 (0.17)^a,c^	1.14 (0.16)^c^	** < 0.001**
Lateral knee joint contact force 2nd half of stance	1.11 (0.29)^b^	0.90 (0.29)^c^	1.40 (0.23)^b,c^	** < 0.001**

Normally distributed data: Mean with standard deviation in parenthesis. *Not normally distributed data: Median with interquartile range in parenthesis. MFA: mechanical femorotibial angle (non-invasive marker-based approach^34^), varus malalignment is depicted as positive angles and valgus malalignment as negative angles.

Significant values are in bold.

^a^Significant difference no rebound vs. rebound.

^b^Significant differences no rebound vs. controls.

^c^Significant differences rebound vs. controls.

At the time of explantation, the external KAM2 and the KAM impulse were significantly higher in both patient groups compared to controls. The peak medial knee joint contact forces did not show any significant group differences. In contrast, the peak lateral knee joint contact force in the first half of the stance phase was significantly lower in the rebound group compared to the no rebound group and controls. The peak lateral knee joint contact force in the second half of the stance phase was significantly lower in both patient groups compared to controls (Table 2).

For the binary logistic regression analysis (Table 3), only independent variables, available at the time of implantation or explantation, that allowed significant differentiation between groups (no rebound, rebound) based on the aforementioned analyses were included. The two most important predictors for rebound were as follows: 1. reduced peak lateral knee joint contact force in the first half of the stance phase at the time of explantation (model 1, 72.7% accurate prediction); 2. minor initial deformity according to the MFA (model 2, 70.5% accurate prediction). A stepwise forward method of regression (likelihood ratio method) including both variables slightly but significantly increased the prediction accuracy to 75.0% (model 3). The addition of the variables peak lateral knee joint contact force during the first and second half of the stance phase at the time of implantation, as well as age and BMI (implantation and explantation) did not significantly improve prediction accuracy of this model. Other potential predictors (indicating significant differences between no rebound and rebound) showed high collinearity with the included variables (MAD with MFA: *r* = 0.927, *p* < 0.001), or were not available at the time of implantation or explantation (residual growth from the time of explantation to the one-year follow-up).

**Table 3:** Binary logistic regression analysis.

Model	Included variables	95% CI for exp *b*				Rebound prediction
		*B (SE)*	Lower	exp *b*	Upper	
1	Constant	3.911* (1.643)				
	latKCF1_expl [N/(kg × m/s^2^)]	− 4.542* (1.773)	0.000	0.011	0.344	72.7%
2	Constant	2.794* (1.240)				
	MFA_impl [°]	0.524* (0.207)	1.124	1.688	2.535	70.5%
3	Constant	7.107** (2.332)				
	latKCF1_expl [N/(kg × m/s^2^)]	− 4.606* (1.897)	0.000	0.010	0.412	75.0%
	MFA_impl [°]	0.537* (0.224)	1.102	1.710	2.653	

A stepwise forward method of regression (likelihood ratio method) was performed in all 50 patients. The following predictor variables that allowed a significant differentiation between groups (no rebound, rebound) were included: 1. peak lateral knee joint contact force during the first half of the stance phase at the time of explantation (latKCF1_expl); 2. mechanical femorotibial angle at the time of implantation of the plates (MFA_impl). The variable peak lateral knee joint contact force during the first half of the stance phase at the time of implantation did not significantly improve prediction accuracy of model 3.

Model 1 with latKCF1_expl as included variable: R^2^ = 0.170 (Cox & Snell), 0.228 (Nagelkerke). Model X^2^ (1) = 8.183, *p* = 0.004.

Model 2 with MFA_impl as included variable: R^2^ = 0.166 (Cox & Snell), 0.223 (Nagelkerke). Model X^2^ (2) = 8.000, *p* = 0.005.

Model 3 with latKCF_expl and MFA_impl as included variables: R^2^ = 0.293 (Cox & Snell), 0.393 (Nagelkerke). Model X^2^ (3) = 15.260, *p* < 0.001.

B, Coefficient of regression. SE, Standard error. *p < 0.05. **p < 0.01. ***p < 0.001.

### Study population characteristics

There were no significant differences in age, body height, and BMI among the two patient groups at the time of implantation and explantation. In contrast, the residual growth (change in body height) from the time of explantation to the one-year follow-up was significantly higher (*p* = 0.009) in the rebound group (Table 1).

More male (66%) than female (34%) patients were included in this study. However, there were no sex differences between the two groups (no rebound, rebound, *p* = 0.797) (Table 1). In 35 of 50 patients (70%) plates were implanted on the medial distal femur, in 7 patients (14%) on the medial proximal tibia, and in 8 patients (16%) on the medial distal femur and the medial proximal tibia. There was no significant difference in the distribution of implant localization between these two patient groups (*p* = 0.347) (Table 1). The average implantation period was 12.1 ± 5.1 months in the group without a rebound and 9.9 ± 2.4 months in the rebound group, without any significant difference (*p* = 0.068). The longitudinal change of body height between implantation and explantation of the plates was also not significantly different between both groups (*p* = 0.818) (Table 1).

The three groups did not show any significant differences in sex and age neither at the time of implantation nor at the time of explantation (Table 2). At both times, BMI was significantly higher in both patient groups compared to controls, and controls had a significantly shorter body height than the no rebound group. The non-invasive marker-based MFA calculated using the static gait analysis trial did not show significant group differences or pathological values at the time of explantation (Table 2).

## Discussion

The high rebound rate of 44% is in accordance with the study of Ko et al.^16^. (42.6% rebound rate) and clearly highlights a clinical problem after initial successful TH in children and adolescents with idiopathic knee valgus malalignment that has to be seriously considered and to be explained to the parents. In the present study, we focused on predictors that are available at the time just before a possible implantation or explantation and thus may influence the indication for a TH, or the timing of implant removal. Two crucial predictors of rebound were identified: 1. reduced peak lateral knee joint contact force in the first half of the stance phase at the time of explantation; 2. minor initial deformity according to the MFA. The best rebound prediction (75.0%) was obtained by including both parameters in the regression method. In contrast, age and BMI did not play a critical role in rebound development.

### Influence of dynamic knee joint loading on rebound prediction

The mechanical load at the knee joint during gait can affect the development of the leg axis^10,11^. The KAM is a commonly used surrogate measure for medial compartment knee loading because it is statistically associated with OA initiation and progression^13,14^. Although conventional knee joint moments during gait did not play a critical role as rebound predictor in our patient group, they can be used as a decision criterion for the indication of TH^17^. In particular, the indication for a TH should be considered when the knee joint moments in the frontal plane are significantly lower compared to healthy controls at the same age. This was only the case in the patient group without a rebound incident (Table 2). In contrast, patients with frontal plane knee joint moments that were only slightly and not significantly different compared to controls (rebound group) appeared to be at increased risk for a rebound. In clinical practice, a pathological joint load is assumed if the deviation of the knee joint moments in the frontal plane exceeds one times the standard deviation of an age-matched reference group^17^.

Calculating joint contact forces requires additional use of musculoskeletal simulation software (e.g., OpenSim). Joint contact forces are part of the internal load and are mainly generated by muscles during walking^18,19^ and thus may be more representative of cartilage loading^20^. At the time of explantation, the peak lateral knee joint contact force in the first half of the stance phase was significantly reduced in patients with a rebound incident compared to patients with no rebound incident and controls in the present study. The exact mechanisms by which growth plate responds to mechanical loading remain largely unclear^21^, although systemic, local, and mechanical factors play a role^22^. Mechanically, mild tension and compression encourage longitudinal bone growth, whereas compression loads above or below a certain threshold inhibit longitudinal bone growth^10^. Therefore, bone growth has a nonlinear relationship with mechanical loading. When this is applied to our results, it may be assumed that the pathological reduction of the peak lateral knee joint contact force in the rebound group led to growth inhibition in the lateral part of the growth plate, resulting in a recurrent genu valgum deformity. In contrast, the peak medial knee joint contact forces were not significantly different between the groups and could therefore be considered physiological, which is related to normal bone growth in the medial part of the growth plate. Therefore, in our opinion, this incongruity in the knee joint at possible explantation can contribute to the development of a rebound. These group differences could not be attributed to the effect of sex, age, walking speed, or other spatiotemporal gait parameters, as no differences between groups exist in these parameters. In addition, the static leg axis parameters (MAD and MFA) did not show significant group differences or pathological values at the time of explantation. This further indicates firstly, the plates were removed when the leg axis was aligned (no overcorrection were performed), and secondly, the control group did not present leg axis deformities.

Some authors have recommended overcorrection to overcome the rebound phenomenon, without suggesting a specific procedure^23,24^. Our study provides evidence that patients with a pathological reduction of peak lateral knee joint contact force at the time of possible explantation may benefit from this strategy, but it remains controversial as there are no established guidelines for the amount of overcorrection to be performed^5^. Therefore, it is recommended that the amount of overcorrection should be limited to achieve acceptable alignment without causing an opposing deformity (genu varum). Additionally, it is still unclear whether overcorrection can prevent deformity recurrence.

### Influence of static leg axis alignment on rebound prediction

Unlike the study by Leveille et al.^5^, a more severe preoperative deformity was not a risk factor for rebound in our study. However, the deformities in that study were pronounced (> 20° mechanical axis deviation from neutral) and cannot be compared directly with our patient group. Zaidman et al.^25^ also did not find a positive correlation between the magnitude of initial deformity and rebound. The results of the present study suggest that a minor initial deformity with a MFA of approximately 5° or less was more predictive of a rebound than a more severe initial deformity. This radiological examination goes hand in hand with the aforementioned physiological dynamic frontal plane knee joint moments in the group with a rebound incident. Considering the high rebound rate in patients with idiopathic knee valgus malalignment, we recommend the indication for a TH when the axial malalignment (MFA) exceeds 5°, and/or the MAD falls within zone 2 or 3 according to the classification originally developed by Müller and Müller-Färber^26^. Additionally, for unclear or borderline cases with marginal deviations in MAD (zones 1 and 2) and MFA, instrumented gait analysis and the determination of dynamic knee joint loading^17,27^ should be included to determine whether surgical treatment is necessary.

### Influence of anthropometric parameters on rebound prediction

In the field of academic writing, a common risk factor for rebound is a young age at the beginning of treatment with high remaining growth potential after implant removal^5–8^. Although age difference between groups was not significant in our study, the residual growth from implant removal to the one-year follow-up was significantly higher in the rebound group. Therefore, accurately estimating skeletal maturity and predicting residual growth are crucial for determining the optimal timing for growth-guiding interventions. Various methods exist for determining remaining growth potential and optimal timing for epiphysiodesis procedures, but their accuracy is discussed controversially, and their applicability in specific patient populations has not been extensively studied^28–30^. Additionally, the presence of residual growth alone is not the only factor the determines the possibility of rebound, as recurrence of the initial deformity can occur in adulthood after axis correction following high tibial osteotomy^9^.

Based on our homogeneous patient group with idiopathic knee valgus malalignment, a higher^6^ or lower^7^ BMI did not play a critical role in the development of a rebound. However, both patient groups showed a significantly higher BMI compared to healthy controls at the same age. As the calculation of the dynamic joint loading was normalized to body mass in the present study, this difference has no influence on our gait analysis results. A strong association between higher BMI and knee valgus malalignment has previously been shown^31,32^.

### Limitations

The results of the present study should be interpreted in the light of its limitations. First, although the dynamic knee joint moments and contact forces are validated mathematical calculations of the mechanical loading of the knee joint, they do not investigate how these global loads translate into soft tissue loads in the growth plate. In a further methodological step, the finite element method could provide a deeper understanding of locally varying mechanical loading of tissue regions with complex, irregular geometries and their dependency on external loads and boundary conditions. Using the finite element method, it is possible to investigate how tension-band plates change the distribution, type, and size of mechanical stresses in juvenile growth plates^33^. Second, we included more male (66%) than female (34%) participants. Although we did not detect significant differences in sex between groups, potential differences in sex regarding rebound prediction should be investigated in a larger study population with a balanced proportion of female and male participants.

## Conclusion

In conclusion, a substantial rebound rate of 44% following successful surgery with TH in children and adolescents with idiopathic knee valgus malalignment highlights the importance of regular follow-up for these patients, ideally until skeletal maturity. To minimize radiation exposure during examinations, a non-invasive marker-based approach using instrumented gait analysis can be considered if available^34^. This study identified two key predictors of the rebound phenomenon: 1. reduced peak lateral knee joint contact force in the first half of the stance phase at the time of explantation (72.7% prediction); 2. minor initial deformity with an MFA of approximately 5° or less (70.5% prediction). The best prediction (75%) was obtained by incorporating both parameters in the regression method. This is the first study emphasizing the importance of the dynamic load situation at the knee joint for optimized treatment of children and adolescents with idiopathic knee valgus malalignment. A TH should not be recommended for patients with physiological frontal plane knee joint moments and a minor deformity of the leg axis. At the time of possible explantation, instrumented gait analysis in combination with MSK modeling can be used to determine whether the plates should be removed or remain in the joint to achieve a slight overcorrection.

## Materials and methods

### Participants/study design

In this prospective cohort study (level of evidence: III), 50 children and adolescents with idiopathic knee valgus malalignment without other orthopedic comorbidities and an indication for TH were evaluated at three different time points (a few days before implantation, a few days before explantation, and one year after removal of the plates) between August 2018 and July 2023 (Table 1). The indication for TH was set for skeletally immature patients with a pathological idiopathic valgus alignment deformity (mechanical axis deviation (MAD) of > 10 mm lateral and/or mechanical femorotibial angle (MFA) of > 3° valgus) of one or both lower extremities based on a full-length standing anteroposterior radiograph^35^. The decision for implant insertion in the femur, tibia, or both segments was made according to pathological joint surface angles: mechanical lateral distal femur angle (mLDFA) and mechanical medial proximal tibia angle (mMPTA) (physiological values for mLDFA 88 ± 2.5° and for mMPTA 87 ± 2.5°)^36^. In the case of bilateral involvement, only the more severely affected leg was analyzed. Either Eight-Plates (Orthofix, Lewisville, TX, USA) or Pedi-Plates (Orthopediatrics Inc., Warsaw, IN, USA) were used as implant. A deviation of the MFA of ≥ 3° into valgus between explantation and the one-year follow-up period was chosen to classify a rebound^7,16^. This means, if the MFA increased by ≥ 3° into valgus (independent of the absolute value of the leg axis), it is assumed as rebound. A one-year follow-up period was chosen because a temporary over-activation of growth has been observed for a period between eight to ten months following implant removal^37^. In addition, it has been shown that recurrence of deformity typically occurred within one year after plate removal^38^. Patients were excluded if they had rheumatoid arthritis, neuromuscular disorders, bony displasias, sagittal or transverse plane deformities of the leg tested by clinical examination, leg length discrepancy of more than 1 cm, clubfoot, flatfoot deformity receiving corrective surgery, history of major trauma of the lower extremity.

Fifteen typically developing, healthy children and adolescents at the same age were recruited as control group for the gait analysis data (Table 2). Only one leg was randomly chosen to be included in the analyses. None of the enrolled individuals reported ankle, knee, hip, or back pain that required treatment at the time of measurements. All participants and their parents were familiarized with the study protocol. The participants and their parents provided written informed consent to participate in this study, as approved by the local ethics committee of the Faculty of Medicine at the Goethe University Frankfurt/Main (number: 182/16) and in accordance with the Helsinki Declaration. This study was first registered in the German Clinical Trials Register (DRKS) (number: DRKS00010296) on 21/04/2016.

### Radiographic evaluations

MAD and MFA were determined on a full-length standing anteroposterior radiograph with TraumaCad® (version 2.3.4.1, Voyant Health, Petach-Tikva, Israel). The criteria for a valid radiographic image were: patient standing in a weight-bearing position, both legs parallel to each other and shoulder-width apart, fully extended knees, and patella centered over the femoral condyles pointing straight forward to avoid rotational errors^39^.

The malalignment analysis was performed following the principles described by Paley et al.^36^. The MFA was defined as the angle formed by the line from the center of the hip to the center of the knee (mechanical femur line) and the line from the center of the knee to the center of the ankle (mechanical tibia line)^40^. The center of the hip joint was determined by drawing a best-fitting circle around the head of the femur using the tools provided in TraumaCad®. The center of the knee joint was defined as the midpoint between the center of the intercondylar region and the center of the eminentia intercondylaris. The center of the ankle was defined as the midpoint of the tibial articular surface of the talus. Neutral alignment was defined as 0°, varus malalignment as positive angle, and valgus malalignment as negative angle.

The MAD was defined as the distance between the center of the knee and the mechanical axis of the limb, with medial deviation (varus) depicted as positive and lateral deviation (valgus) as negative values. Intra- and interobserver reliability was excellent for MFA (intraobserver reliability: ICC_2,1_ = 0.974; 95% CI 0.963–0.981; interobserver reliability: ICC_2,1_ = 0.982; 95% CI 0.972–0.989) and MAD (intraobserver reliability: ICC_2,1_ = 0.994; 95% CI 0.992 − 0.996; interobserver reliability: ICC_2,1_ = 0.995; 95% CI 0.993–0.996) measurements^41^.

### Gait analysis methods

Kinematic data were collected at 200 Hz using an 8-camera motion capture system (MX T10, VICON Motion Systems, Oxford, UK). Ground reaction forces were recorded at 1000 Hz using two force plates (Advanced Mechanical Technology, Inc., Watertown, MA, USA) embedded at the midpoint of a 15 m long level walkway. For each subject and time point, a series of five barefoot walking trials at a self-selected speed were averaged for further analysis on the basis of complete marker trajectories and a clear foot-forceplate contact. For enhanced reliability and accuracy, a lower body protocol (MA), described in a previous investigation, was used^42^. In addition to the standardized Plug-in-Gait marker set^43^, reflective markers on the medial malleolus, medial femoral condyle, and greater trochanter were used to determine the centers of rotation for the ankle, knee, and hip joints. The centers of rotation for the ankle and knee joints were defined statically as the midpoints between the medial and lateral malleolus and femoral condyle markers. The center of the hip joint was calculated using a standardized geometrical prediction method using regression equations^44^.

External knee joint moments (normalized to body mass) were determined using an inverse dynamics approach^44^. The distinctive “m” or “double hump” shape of the external knee adduction moment (KAM) waveform in the frontal plane has led researchers to report several discrete variables related to it^15,45^. Therefore, the first (KAM1) and second peaks (KAM2) of the knee adduction moment in loading response/mid stance and terminal stance were analyzed. In addition, the impulse of the KAM (area under the curve)^46^ was calculated. To account for possible differences in leg length, walking speed and step length were normalized to leg length according to Hof^47^.

### Musculoskeletal modeling

Input from marker positions and ground reaction forces were prepared using the MOtoNMS toolbox (version 3) in MATLAB (2020b, The MathWorks, Inc., Natick, MA, USA) for use in OpenSim (4.1)^48^. Force data were filtered using a zero-lag low-pass Butterworth filter with a cut-off frequency of 10 Hz. An OpenSim model^18^ with 20 degrees of freedom was used. The knee joint had sagittal and frontal-plane rotational degrees of freedom, and the medial and lateral contact forces were resolved using a multi-compartment knee model^19,49^. We further personalized the models by adjusting the frontal-plane alignment with the MFA measured from X-ray images^19^. X-rays were not available for the control group, and the MFA was calculated using a static gait analysis trial^27,34^. This non-invasive marker-based approach correlates well with the determination of lower limb alignment in the frontal plane using radiographs in young patients with varus or valgus malalignment^34^. Peak knee contact forces in loading response/mid stance and terminal stance were normalized by body mass and computed as the reaction force in the medial and lateral compartments of the knee in the direction of the long axis of the tibia^27,50^.

### Statistical analysis

Statistical analyses were performed using SPSS Statistics (version 29, IBM Corporation, New York, NY, USA). The Shapiro–Wilk test was used to verify normal distribution. All variables were normally distributed, except residual growth from the time of explantation to the one-year follow-up, BMI and step width at the time of implantation and explantation, as well as the following variables at the time of implantation: peak KAM in the first half of the stance phase, and peak medial knee joint contact forces in the first and second half of the stance phase.

Differences in anthropometric and clinical data between both patient groups (no rebound, rebound) were tested for significance using an unpaired, two-tailed Student’s *t*-test for normally distributed data and a Mann–Whitney U-test for non-normally distributed data. A chi-squared test was used to compare the sex distribution and the implant localization between groups.

Group differences (no rebound, rebound, controls) including gait data were derived from one-way ANOVA for normally distributed data. In case of significance, unpaired two-tailed post-hoc *t*-tests with Bonferroni correction were performed to control for type 1 errors. A Kruskal–Wallis test following a Mann–Whitney post-hoc test in case of significance were used for not normally distributed data.

A binary logistic regression analysis was performed to determine predictor variables that best explained the rebound phenomenon. In particular, a stepwise forward method of regression (likelihood ratio method) was performed by setting the occurrence (yes or no) of the rebound phenomenon as a dependent variable. For this purpose, only independent variables that allow a significant differentiation between groups (no rebound, rebound) at the time of implantation and explantation based on the previously described analysis above were included. Multicollinearity (*r* > 0.7) between potential predictor variables was excluded^51^. The significance level was set at *p* ≤ 0.05.

## Data Availability

The datasets generated and analyzed during the current study are available from the corresponding author on reasonable request.
